# A QUBO Formulation of the Stereo Matching Problem for D-Wave Quantum Annealers

**DOI:** 10.3390/e20100786

**Published:** 2018-10-12

**Authors:** William Cruz-Santos, Salvador E. Venegas-Andraca, Marco Lanzagorta

**Affiliations:** 1 CU-UAEM Valle de Chalco, Hermenegildo Galeana 3, Valle de Chalco 56615, Estado de México, Mexico; 2 Tecnologico de Monterrey, Escuela de Ingenieria y Ciencias. Ave., Eugenio Garza Sada 2501, Monterrey 64849, NL, Mexico; 3 US Naval Research Laboratory, 4555 Overlook Ave., SW Washington, DC 20375, USA

**Keywords:** quantum annealing, stereo matching, quantum algorithms

## Abstract

In this paper, we propose a methodology to solve the stereo matching problem through quantum annealing optimization. Our proposal takes advantage of the existing Min-Cut/Max-Flow network formulation of computer vision problems. Based on this network formulation, we construct a quadratic pseudo-Boolean function and then optimize it through the use of the D-Wave quantum annealing technology. Experimental validation using two kinds of stereo pair of images, random dot stereograms and gray-scale, shows that our methodology is effective.

## 1. Introduction

Computer vision is an interdisciplinary field of research with almost six decades of theoretical and algorithmic developments [1,2] that focuses on developing mathematical techniques and algorithms that aim at enabling computers to identify, analyze, and understand information from elements of imagery [3]. Computer vision has many links and common interests with Artificial Intelligence and Machine Learning.

Stereo vision refers to the ability to extract information about the 3-D structure and distance of a scene from two or more images taken from different viewpoints. The most basic stereo system consists of two cameras (left and right) and any stereo system must solve two problems:

1. The stereo matching problem [4]: Which parts of the left and right images are projections of the same scene element?
2. The reconstruction problem, which is stated as follows: given a number of corresponding parts of the left and right images, what can we say about the 3-D locations and structures of the observed objects?

In this paper, we focus on the stereo matching problem using a basic stereo system. This problem is difficult to solve because some parts of the scene are visible only by either the left or right camera but not by both; therefore, a stereo system must also be able to select the image parts to be matched [5]. Stereo vision has many applications, such as in photogrammetry [6], stereo-based head tracking [7], volumetric and 3-D surface reconstruction [8], video-based walkthroughs [9], and stereo-based autonomous navigation [10], among others.

Nowadays, there is an increasing amount of interest in applying quantum computing techniques on machine learning tasks. The research area of quantum machine learning (QML) [11,12] has as its main objective designing quantum algorithms and data representation using quantum states that outperform classical algorithms in machine learning problems. Examples of recent progress in QML are the proposals of quantum principal component analysis of classical data [13] and quantum support vector machine [14] (both papers use quantum states that encode classical data as input), as well as deep quantum learning [15] and sampling of a quantum Boltzman machine [16] (these two papers have classical data as input). More recent advances in QML can be found in [17,18].

Quantum hardware technologies such as the quantum circuit-based IBMQ computer [19] and the D-Wave [20] quantum annealer machine have motivated the development of applications in QML. In particular, the D-Wave computer has evolved and grown significantly with respect to the number of qubits, allowing the posing of problems of interest [21,22,23,24]. For instance, the current generation of D-Wave 2000Q processors has almost 2048 working superconducting qubits connected in a graph topology known as Chimera.

In this paper, we present a methodology for writing a Quadratic Unconstrained Binary Optimization (QUBO) formulation of the stereo matching problem via a graph-cut approach, followed by using this QUBO formulation to simulate a quantum annealing algorithm on D-Wave’s simulation software. This paper is meant to be a contribution to the nascent area of quantum algorithms to solve relevant problems in science, engineering and other fields, like finance.

Our method could also be used to implement NP-hard problem instances in novel architectures of classical hardware, like the Ising model architectures proposed by Fujitsu [25] and Hitachi [26] research laboratories (examples of NP-hard problems are combinatorial optimization problems. A concise introduction to NP-hardness can be found in [27]). Furthermore, since the stereo matching problem is of paramount importance in computer vision, several classical algorithms have been developed to solve it, among them, graph-cut-based methods (we provide a brief account of algorithms for stereo matching in Section 2). Although the stereo matching problem is in the P class, a minimum upper bound for the computational complexity is unknown; hence, the development of novel approaches is pertinent. In this sense, the proposal presented in this manuscript combined with present and future research on quantum annealing-based algorithms could be used in future work to develop new and less expensive algorithms.

Our work consists of two main goals: (a) to express a problem of interest as a QUBO problem and (b) to find a minor embedding of the QUBO problem into the Chimera graph architecture. Before applying our methodology, we represent the stereo matching problem as a graph-cut problem by constructing an edge weighted graph with two special vertices. This weighted graph has the following property: finding a subset of edges with minimum cost whose removal disconnects the special vertices is equivalent to solving the stereo matching problem. Therefore, for step (a), we construct a pseudo-Boolean expression of degree three for the graph-cut problem, and we use a reduction method to obtain a QUBO expression to be minimized. For step (b), a minor embedding is found by using the software tools provided by the D-Wave solver application programming interface (SAPI). To validate our methodology for the solution to the stereo matching problem, we present the results of the minimization of the QUBO expression using the classic solver qbsolv developed by D-Wave.

This paper is organized as follows: In Section 2, we succinctly introduce the basic notions of optimization via quantum annealing and we describe our methodology for solving the stereo matching problem using QUBO and D-Wave technology; in Section 3, we present our experiments and results; finally, we present our conclusions in Section 4.

## 2. Results

### 2.1. Quantum Annealing

Quantum annealing (QA) [28,29,30,31,32,33] can be seen as a quantum version of Classical Simulated Annealing (SA) [32,33,34,35]. SA uses the process of annealing in which a solid in a thermal bath is heated up by increasing the temperature, followed by a cooling by slowly lowering the temperature of the bath. The annealing allows the particles in the solid to go from a random configuration or high energy to a lattice configuration or low energy. This process was found to be equivalent to the problem of finding the configuration with the minimum amount of energy or the cost function of a given combinatorial optimization problem. QA employs quantum fluctuations to anneal the system down to its minimum energy state [33]. The most used physical system for QA is the Ising spin glass model on *N* particles described by

(1) $$H_{p}=\sum_{1\leq i\leq N}h_{i}\sigma_{i}^{z}+\sum_{1\leq i<j\leq N}J_{ij}\sigma_{i}^{z}\sigma_{j}^{z}$$

where $\sigma_{i}^{z}$ is the Pauli matrix *z* acting on particle *i*, $h_{i}$ describes the magnetic field on particle *i*, and $J_{ij}$ is the coupling strength between particles *i* and *j*. The minimum energy state or ground state of the Hamiltonian $H_{p}$ corresponds to a configuration $s=\left(s_{1},\cdots ,s_{N}\right)\in {\left\{+1,-1\right\}}^{N}$ of spins that minimizes the following energy function

(2) $$E\left(s\right)=\sum_{1\leq i\leq N}h_{i}s_{i}+\sum_{1\leq i<j\leq N}J_{ij}s_{i}s_{j}.$$

It is known that the problem of finding a configuration $s^{*}$ with minimum energy $E(s^{*})$ is an NP-complete problem [36] (although some special cases of the Ising model can be solved in polynomial time. However, those cases are not part of our study). A related problem with the same complexity is obtained by changing the variable $x_{i}=\left(1+s_{i}\right)/2$ for i=1,⋯,N. In other words, finding a configuration $x\in {\left\{0,1\right\}}^{N}$ such as E(x) that is minimum also corresponds to an NP-complete problem [37].

One of the schemes to realize QA is through adiabatic quantum evolution from the ground state of an initial Hamiltonian to the ground state of a final Hamiltonian which corresponds to the solution of a given problem [38]. According to this scheme, a time-dependent Hamiltonian takes the form

(3) $$H\left(\tau \right)=A\left(\tau \right)H_{0}+B\left(\tau \right)H_{p}$$

where $\tau =t/t_{a}$ for $0\leq t\leq t_{a}$, is $t_{a}$ is the total annealing time, and the initial Hamiltonian $H_{0}=-\sum_{1\leq i\leq N}\sigma_{i}^{x}$ is responsible for quantum tunnelling among the localized classical states corresponding to the eigenstates of the Hamiltonian $H_{p}$. In the current generation of the D-Wave 2000Q processor, functions A(τ) and B(τ) are defined so that at time τ=0, the influence of the Hamiltonian $H_{0}$ is predominant against $H_{p}$. As time evolves from τ=0 to τ=1, the influence of the Hamiltonian $H_{p}$ increases, while $H_{0}$ fades away.

One then can solve the time-dependent Schrödinger equation with the Hamiltonian H(t) to obtain an approximate solution to the dynamics of the system. Consider the Hamiltonian $H\left(t\right)=\tilde{H}\left(t/t_{a}\right)=\tilde{H}\left(\tau \right)$ such that 0≤τ≤1, and let us denote by |l;τ〉 the instantaneous eigenvector of $\tilde{H}\left(\tau \right)$ corresponding to the instantaneous eigenvalue $\lambda_{l}\left(\tau \right)$. Then,

(4) $$\tilde{H}\left(\tau \right)|l;\tau \rangle =\lambda_{l}\left(\tau \right)|l;\tau \rangle$$

with

(5) $$\lambda_{0}\left(\tau \right)\leq \lambda_{1}\left(\tau \right)\leq \cdots \leq \lambda_{2^{N}-1}\left(\tau \right).$$

The Adiabatic theorem asserts that for sufficiently large $t_{a}$,

(6) $$\lim_{t_{a}\to \infty }\left|\langle l=0;\tau =1|\psi \left(t_{a}\right)\rangle \right|=1$$

for some solution ψ(t) to the Schrödinger equation with the Hamiltonian H(t). Consequently, the state $\psi (t_{a})$ will be very close to the ground state of the Hamiltonian $H_{p}$ with a high probability. A sufficient condition for the algorithm running time that is needed to satisfy the Adiabatic theorem is

(7) $$t_{a}\gg \frac{\varepsilon_{max}}{g_{min}^{2}}$$

where

(8) $$g_{min}=\min_{0\leq \tau \leq 1}\left(\lambda_{1}\left(\tau \right)-\lambda_{0}\left(\tau \right)\right)$$

and

(9) $$\varepsilon_{max}=\max_{0\leq \tau \leq 1}\left|\langle l=1;\tau \right|\frac{d\tilde{H}}{d\tau }|l=0;\tau \rangle |.$$

A more rigorous formulation of the Adiabatic theorem can be found in [39]. It must be noted that in realistic physical settings, including the D-Wave processor, the QA dynamics of a system do not necessary evolve adiabatically. There are several reasons including thermal fluctuations and additive noise from the environment. Other considerations to take into account when solving problems on a D-Wave processor are the size of the device or number of qubits in the current architecture and the precision of the Ising parameters, such as the magnetic field values $h_{j}$ and the coupler strength $J_{ij}$ between pairs of qubits. A concise introduction to quantum annealing algorithms, their potential relevance for the advancement of key problems in theoretical computer science, as well as mathematical methods to implement quantum annealing algorithms in D-Wave quantum annealers can be found in  [27].

#### 2.1.1. QUBO Formulation Approach to the Stereo Matching Problem

The stereo matching problem is of key importance in computer vision [5,40]. Without any loss of generality, a stereo setup consists of two spatially separated cameras located at the same height with parallel optical axes. A stereo pair of images, $I_{l}$ and $I_{r}$, are the recorded images by the left and right cameras, $c_{l}$ and $c_{r}$, respectively. For a point *P* in a scene observed by a stereo setup, it (point *P*) will be projected into the left and right images with intensities $I_{l}\left(x_{1},y\right)$ and $I_{r}\left(x_{2},y\right)$, respectively. Notice that, in a stereo setup, a point *P* will be projected at the same row *y* in both left and right images, whilst determining the columns $x_{1}$ and $x_{2}$ of projection is a difficult problem [4]. Let us now state the following problem.

**Figure 1:**
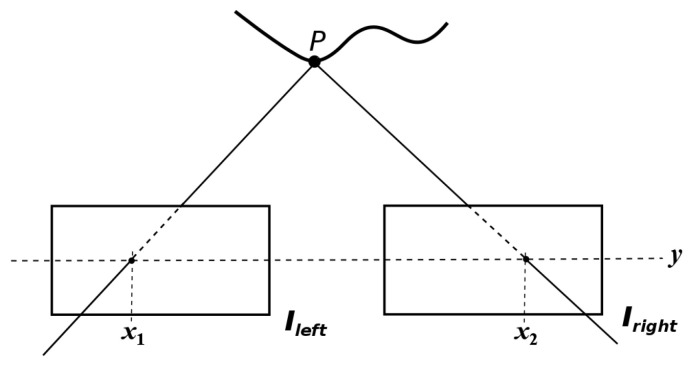
Example of the projection of a point *P* on the image planes of the left and right images. As can be seen in this illustration, the point *P* is projected to the positions $x_{1}$ and $x_{2}$ of the left and right images, respectively. The difference $x_{2}-x_{1}$ is called the disparity of point *P*.

**Problem** **1** (Stereo matching)**.** *Given a stereo pair of images $\left(I_{l},I_{r}\right)$, find, for every pixel $I_{l}\left(x_{1},y\right)$ in the left image, its corresponding projection $I_{r}\left(x_{2},y\right)$ in the right image (see Figure 1).*

The solution to problem 1 involves calculating the disparity for every pixel in the left image. If the calibration parameters of the stereo setup are given [40], then it is possible to infer the depth at every point in the scene. Early approaches to solve problem 1 are those based on block matching techniques [4], dynamic programming [41], belief propagation [42] and graph-cut [43] based techniques, among others. In the following section, we formulate the stereo matching problems in terms of graph-cuts.

#### 2.1.2. Energy Function

Let P be the image domain or set of pixels in the left image and let L be the set of labels or disparities; a labeling is a map

(10) l:P→L

such that for each pixel p∈P, *l* assigns a label $l\left(p\right)=l_{p}$ to the pixel *p*.

Given a labeling *l*, its cost is defined as

(11) $$E\left(l\right)=\sum_{p\in P}D_{p}\left(l_{p}\right)+\sum_{\{p,q\}\in N}V_{pq}\left(l_{p},l_{q}\right)$$

where $D_{p}$ models the cost of assigning label $l_{p}$ to the pixel *p*, $V_{pq}$ models the cost of assigning $l_{p}$ to *p* and $l_{q}$ to *q*, with *p* and *q* being adjacent pixels, and N is the set of neighbor pairs {p,q}.

Graph-cut techniques consist of constructing a graph with weighted edges and two special vertices, *s* and *t*. In this model, finding a cut with minimum cost that separates *s* and *t* is equivalent to minimizing function E(l) [44,45].

#### 2.1.3. Graph Construction

The following graph construction is based on the Boykov and Kolmogorov approach for Min-Cut/Max-Flow Algorithms for Energy Minimization [44].

Let G=(V,E,c) be an edge weighted graph where *V* is the set of vertices with two special vertices, the source *s* and the sink *t*, *E* is the set of undirected edges, and $c:E\to Z^{+}$ is a function that assigns a positive value to each edge in *E*. A cut in *G* is a set of edges whose removal from *E* separates the source from the sink, and its cost is the sum of their weights. Let L={0,⋯,L} be the set of possible disparities or labels. The graph *G* is constructed as follows:

1. To each pixel *p* in the image, we associate a chain composed of L+2 vertices, say $p_{0},p_{1},\cdots ,p_{L+1}$. These vertices are connected by edges called *t*-links, $\left\{e_{p_{0},p_{1}},e_{p_{1},p_{2}},\cdots ,e_{p_{L},p_{L+1}}\right\}$, where $e_{p_{0},p_{1}}=\left\{p_{0},p_{1}\right\},e_{p_{1},p_{2}}=\left\{p_{1},p_{2}\right\},\cdots ,e_{p_{L},p_{L+1}}=\left\{p_{L},p_{L+1}\right\}$. For a disparity range from 0 to *L* values, and for each pixel *p*, we will have L+2 vertices in the chain and L+1 *t*-links between these vertices; each edge defines a labeling with one specific value. Additionally, there are two *t*-links that connect the first vertex with the source and the last vertex with the sink. Hence, there exist L+3 such edges in the graph for each pixel *p*. The weights of those edges directly depend on the function $D_{p}$, which specifies the cost of applying a specific label to a pixel.
2. To each pair of neighbor pixels, *p* and *q*, there will be links that connect the corresponding chains with edges called *n*-links. For instance, let $p_{0},p_{1},\cdots ,p_{L+1}$ and $q_{0},q_{1},\cdots ,q_{L+1}$ be the chain vertices for two neighbor pixels *p* and *q*; the *n*-links between chains are $e_{p_{0},q_{0}}=\left\{p_{0},q_{0}\right\},e_{p_{1},q_{1}}=\left\{p_{1},q_{1}\right\},\cdots ,e_{p_{L+1},q_{L+1}}=\left\{p_{L+1},q_{L+1}\right\}$. Usually a 4-neighborhood is assumed for every pixel. The weights of these edges should reflect the penalty when assigning different labels to neighboring pixels.

**Figure 2:**
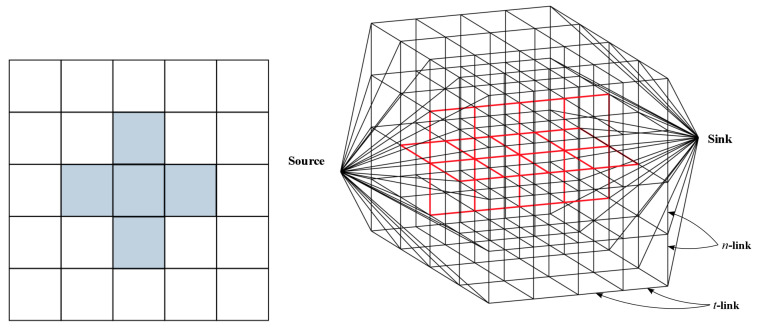
An image of size 5×5 pixels (**left**) and its graph topology (**right**).

For a given stereo pair of images of size N×M pixels and a disparity range *L*, the total number of vertices in the constructed graph is (L+2)NM+2 and the number of edges is NM(L+3)+((M−1)N+(N−1)M)(L+2). In Figure 2, we present an example of the graph topology for an image of 5×5 pixels and L=3, in which we show the *t*-links and *n*-links (red edges) for the central pixel and its neighbor pixels (right). In this example, there are 127 vertices and 350 edges.

A cut separating the source from the sink severs *t*-links as well as *n*-links. The severed *t*-links directly define the labels assigned to each pixel. For instance, if a cut severs the *t*-link $t_{p_{j}}=\left\{p_{j},p_{j+1}\right\}$ with 0≤j≤L for a pixel *p*, then the assigned label to *p* is *j*. Therefore, the cut must sever exactly one *t*-link at every pixel.

The cost of a *t*-link will correspond to the disparity of a given pixel. If the assumption that we have a certain disparity *d* at pixel $p=(x_{1},y)$ is correct, the intensity $I_{l}\left(x,y\right)$ observed in the left camera at position (x,y) should be similar to the intensity $I_{r}\left(x-d,y\right)$ in the right camera observed at position (x−d,y), because both pixels depict the same scene object. Consequently, the cost of a given *t*-link $t_{p_{d}}=\left\{p_{d},p_{d+1}\right\}$ with 0≤d≤L can be set to the $L_{2}$ norm of the intensity difference between corresponding pixels in both camera images:

(12) $$c\left(t_{p_{d}}\right)={\left|I_{l}\left(x,y\right)-I_{r}\left(x-d,y\right)\right|}^{2}+C$$

where *C* is a constant that is chosen to be sufficiently large in order to ensure that exactly one *t*-link is severed by the cut for each pixel. If the intensity difference between $I_{l}\left(x,y\right)$ and $I_{r}\left(x-d,y\right)$ is low enough, this indicates that the corresponding disparity is a good solution and consequently, $c(t_{p_{d}})$ is low. The constant *C* is set to be slightly larger than the sum of the weights of all penalty *n*-links belonging to pixel *p*,

(13) $$C_{p}=1+\left(L-1\right)\sum_{q\in N_{p}}\lambda_{pq}$$

where $N_{p}$ defines the neighborhood of *p*.

The pairwise cost function $V_{pq}$ depends on the difference between labels $l_{p}$ and $l_{q}$. In the simplest case, $V_{pq}$ can be set to the absolute difference $|l_{p}-l_{q}|$. Therefore,

(14) $$V_{pq}\left(l_{p},l_{q}\right)=\lambda_{pq}\left|l_{p}-l_{q}\right|$$

where $\lambda_{pq}$ is a weighting factor for setting the relative importance of the smoothness term, which can be chosen differently for each pair of pixels, but is often set to a constant $\lambda =\lambda_{pq}$ for all p,q∈N. Given an *n*-link $n_{p_{k}q_{k}}$ for two neighbor pixels *p* and *q*, the cost of $n_{p_{k}q_{k}}$ is equal to $c\left(n_{p_{k}q_{k}}\right)=\lambda_{pq}$ for all 1≤k<L.

#### 2.1.4. QUBO Formulation of the Stereo Matching Problem via the Minimum Multicut Problem

**Figure 3:**
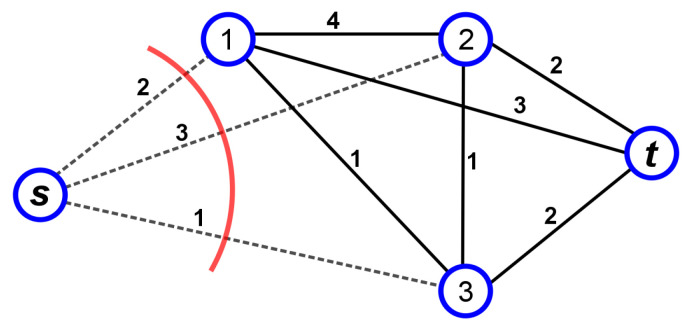
Example of a multi-cut with k=1 for the pair of vertices (s,t). The dotted lines are the edges belonging to the minimum multi-cut and its cost is the sum of their weights.

The graph cut formulation of the stereo matching problem is a special case of the following general problem:

**Problem** **2** (Minimum multi-cut)**.** *Given an edge weighted graph G=(V,E,c) and a set $S=\{\left(s_{1},t_{1}\right),\cdots ,\left(s_{k},t_{k}\right)\}$ of k pairs of vertices, find a multi-cut with minimum cost, i.e., a set $E^{\prime }\subseteq E$ such that the removal of $E^{\prime }$ from E disconnects $s_{i}$ from $t_{i}$ for every pair $(s_{i},t_{i})\in S$, where the cost of $E^{\prime }$ is given as $\sum_{\left\{u,v\right\}\in E^{\prime }}c\left(\left\{u,v\right\}\right)$ (see Figure 3).*

When k=1,2, the minimum multi-cut problem can be solved in polynomial time [46], and it becomes NP-hard for k≥3 for general graphs [47]. In the special case where *G* is restricted to trees and for arbitrary *k*, Problem 2 remains NP-hard [48].

Let us formulate Problem 2 for k=1 as a QUBO expression. Let G=(V,E,c) be a weighted graph and (s,t) be the pair of vertices to be disconnected. For each edge {u,v}∈E, let us associate a Boolean variable $y_{uv}$ such that $y_{uv}=1$ if the edge {u,v} is selected for a cut and $y_{uv}=0$ otherwise. Similarly, for each vertex v∈V, let us associate a Boolean variable $x_{v}$ such that $x_{v}=1$ if v∈U and $x_{v}=0$ in the other cases where *U* is a subset of *V*.

Let $H_{\mathrm{problem}}$ be defined as

(15) $$H_{\mathrm{problem}}=H_{\mathrm{cost}}+H_{\mathrm{penalty}}.$$

The first term, $H_{\mathrm{cost}}$, in Equation (15) expresses the cost of the selected edges to be removed from the graph *G*, and the second term, $H_{\mathrm{penalty}}$, has the purpose of introducing a penalization if the selected edges do not correspond to a cut in *G*.

The term $H_{\mathrm{cost}}$ can be easily defined as

(16) $$H_{\mathrm{cost}}=\sum_{\{u,v\}\in E}y_{uv}\cdot c\left(\left\{u,v\right\}\right)$$

which gives us the cost of the selected edges to be removed.

The second term, $H_{\mathrm{penalty}}$, is constructed as

(17) $$\begin{array}{ccc} H_{\mathrm{penalty}} & = & \alpha [1-x_{s}-x_{t}+2x_{s}x_{t}+\\ & & \sum_{\{u,v\}\in E}\left(1-y_{uv}\right)\left(x_{u}+x_{v}-2x_{u}x_{v}\right)]. \end{array}$$

The penalty term satisfies the criterion that if the selected edges form a cut, then $H_{\mathrm{penalty}}=0$, and $H_{\mathrm{penalty}}>0$ in other cases. The penalty term is constructed based on the observation that the selected edges form a cut if there exists a partition $\left(U,\bar{U}\right)$ with U⊆V such that s∈U and $t\in \bar{U}$, and every edge e∈E with $y_{e}=0$, has its extreme vertices on only one side of the partition, either in *U* or in $\bar{U}$. The coefficient α is a positive value which is used to ensure that solutions that do not constitute a cut are avoided.

The expression $H_{\mathrm{penalty}}$ given in (17) corresponds to a pseudo-Boolean function of the third degree which can be converted into a QUBO expression using a reduction method. Among reduction methods we find the Boros algorithms [37,49,50] and the Freedman [51] and Ishikawa [52] methods. A review of quadratization methods in pseudo-Boolean optimization can be found in [53].

The Ishikawa method says that every positive cubic term $x_{1}x_{2}x_{3}$ can be expressed as a quadratic term with

(18) $$\begin{array}{ccc} x_{1}x_{2}x_{3} & = & x_{1}x_{2}+x_{1}x_{3}+x_{2}x_{3}+\min_{z\in \{0,1\}}z\left(1-x_{1}-x_{2}-x_{3}\right)\\ & \leq & x_{1}x_{2}+x_{1}x_{3}+x_{2}x_{3}+z\left(1-x_{1}-x_{2}-x_{3}\right) \end{array}$$

where *z* is an ancilla Boolean variable. By applying the Ishikawa method to Equation (17) (which is the method that uses the smallest number of ancilla variables to obtain a quadratic expression) we have

(19) $$\begin{array}{ccc} H_{\mathrm{penalty}}^{\mathrm{qubo}} & = & \alpha [1-x_{s}-x_{t}+2x_{s}x_{t}+\sum_{\{u,v\}\in E}(x_{u}+\\ & & x_{v}+2w_{uv}+x_{u}y_{uv}+x_{v}y_{uv}-2x_{u}w_{uv}-\\ & & 2x_{v}w_{uv}-2y_{uv}w_{uv})]. \end{array}$$

where $w_{uv}$ for each {u,v}∈E are ancilla variables.

The final quadratic cost function to be minimized can be written as

(20) $$H_{\mathrm{problem}}^{\mathrm{qubo}}=H_{\mathrm{cost}}+H_{\mathrm{penalty}}^{\mathrm{qubo}}$$

which requires a number 2|E|+|V| of variables to represent the minimum multi-cut problem for a single pair of vertices.

## 3. Experiments and Discussion

In this section, we prove the concept of the solution to the stereo matching problem using the methodology described in Section 2. The following steps summarise our methodology:

(i) Construct the weighted graph described in Section 2.1.3 for the given stereo pair of images.
(ii) Formulate the stereo matching problem as the minimum multi-cut problem for the pair (s,t) using the weighted graph from the previous step.
(iii) Construct the QUBO expression given in Equation (20) from the weighted graph in step (ii) for a single pair.
(iv) Embed into the Chimera graph topology of the D-Wave computer.
(v) Find the minimum energy solution to the QUBO/Ising problem by quantum annealing.

**Figure 4:**
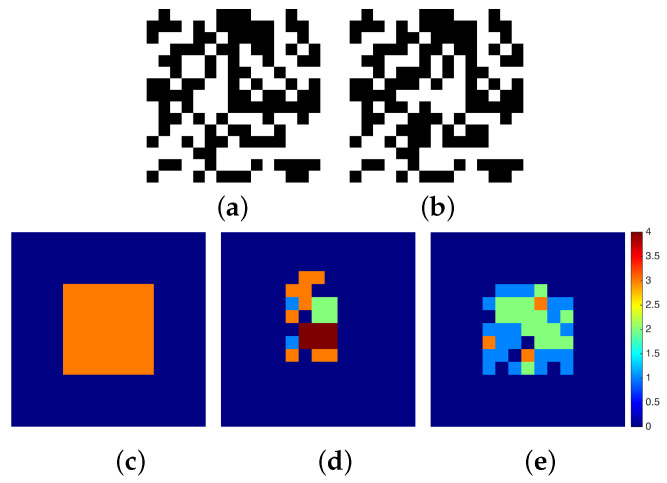
Example of binary random dot stereogram (BRDS) images of size 15 × 15 pixels: (**a**,**b**) left and right BRDS images, (**c**) truth disparity map, (**d**) disparity map obtained using matching techniques, and (**e**) disparity map obtained using our approach.

We consider two case studies of stereo pair images: (a) a binary random dot stereogram (BRDS) and (b) a gray scale imaged object, both with a resolution of 15 × 15 pixels. At this stage of our research, we are unable to study bigger size images because of the large resources needed to represent the stereo matching problem with the current limitations of quantum hardware. In case (a), we generate a BRDS as follows: a left image is created by setting each pixel randomly to either black or white. Then, a copy of this image is made and it is called the right image. Take a region in the right image and shift that region horizontally by *d* pixels to the left. The pixels vacated by shifting the region are filled in with random values. The shifted region simulates an object with a disparity of *d* pixels. In Figure 4 (top), we show a BRDS pair of images with a centered square region of size 7 × 7 pixels shifted by d=3 pixels.

**Figure 5:**
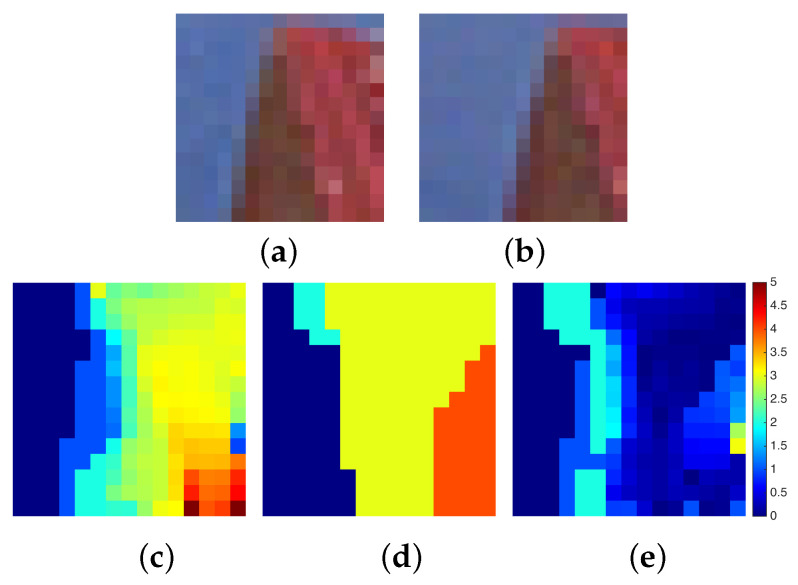
Example of gray scale images of size 15 × 15 pixels: (**a**,**b**) left and right gray scale stereo images, (**c**) disparity map using matching techniques with sub-pixel accuracy, (**d**) disparity map obtained using our approach, and (**e**) map error between the disparity maps (**c**,**d**).

For case (b), we crop a region of size 15 × 15 pixels from a stereo pair of images of a larger size. The cropped images have a maximum disparity range of five pixels. Figure 5 (top) shows a stereo pair showing a corner of an object on a background. In cases (a) and (b), the regions of interest (ROIs) to be matched are of sizes 15×12 and 15×10 pixels, respectively. In Table 1, the size of the weighted graph described in Section 2.1.3 for step (i) in our methodology is shown. Despite the large number of vertices and edges needed to formulate the stereo matching as a graph-cut problem, it is possible to use simplification techniques to limit the volume of the graph [45,54,55].

**Figure 6:**
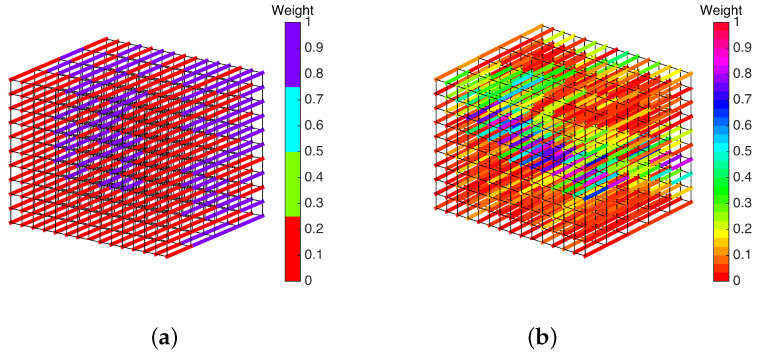
Weighted graphs described in Section 2.1.3 for the (**a**) binary random dot stereogram BRDS and (**b**) gray-scale stereo pair images given in Figure 4 and Figure 5, respectively.

Figure 6 shows the weighted graphs for cases (a) and (b) where the vertices s,t and their incident edges are omitted for better visualization. The *t*-links in the graph, represented by wide lines, are colored according to their weights, and the *n*-links are colored in black as thinner lines. As it can be seen in Figure 6a, we have only two possible weights for a *t*-link because the cost given in (12) for a BRDS can be zero or one plus a constant value.

In the center vertices of the weighted graph, we can see the square region to be matched. In Figure 6a, we show the weighted graph for the gray scale stereo pair given in Figure 5. In this case, there are many possible weights or disparity options. The weights of the omitted *t*-links in Figure 6 are set as high as possible so that they can never be chosen in the multi-cut. In cases (a) and (b), the constant weight λ is chosen in (14) which is equal to *L* for the *n*-links since the disparity is, at most, L−1, and the constant value *C* in (12) is set to be equal to the maximum value of $|I_{l}\left(x,y\right)-I_{r}{\left(x-d,y\right)|}^{2}$ for all possible values from d=0,⋯,L−1 for all pixels.

In step (iii), the stereo matching problem is formulated as a graph-cut problem for a single pair (s,t). For this purpose, a QUBO function is constructed as given in (20). Table 1 presents the number of logical Boolean variables needed to construct the QUBO function for cases (a) and (b). The QUBO function $H_{\mathrm{problem}}^{\mathrm{qubo}}$ can be constructed for the given weighted graphs in Figure 6 as follows:

(21) $$\begin{array}{ccc} H_{\mathrm{problem}}^{\mathrm{qubo}} & = & \sum_{\left\{u,v\right\}\in E_{tlink}}y_{uv}c\left(\left\{u,v\right\}\right)+\sum_{\left\{u,v\right\}\in E_{nlink}}y_{uv}c\left(\left\{u,v\right\}\right)+\alpha \left(1-x_{s}-x_{t}+2x_{s}x_{t}\right)+\\ & & \alpha \sum_{\left\{u,v\right\}\in E_{tlink}}\left(x_{u}+x_{v}+2w_{uv}+x_{u}y_{uv}-2x_{u}w_{uv}-2x_{v}w_{uv}-2y_{uv}w_{uv}\right)+\\ & & \alpha \sum_{\left\{u,v\right\}\in E_{nlink}}\left(x_{u}+x_{v}+2w_{uv}+x_{u}y_{uv}-2x_{u}w_{uv}-2x_{v}w_{uv}-2y_{uv}w_{uv}\right) \end{array}$$

where the set of edges $E=E_{tlink}\cup E_{nlink}$ such that $E_{tlink}$ is the set of *t*-links and $E_{nlink}$ is the set of *n*-links. For the weighted graphs in Figure 6a,b, the number of *n*-links and *t*-links are $|E_{nlink}|=1080$, $|E_{nlink}|=1665$, and $|E_{nlink}|=1200$, $|E_{nlink}|=1925$, respectively.

The coefficient α in (19) that is used to penalize sets of edges that do not correspond to a multi-cut is

(22) $$\alpha =\sum_{\{u,v\}\in E}c\left(\left\{u,v\right\}\right)-\sum_{u\in V}\left[c(\{s,u\})+c(\{t,u\})\right]$$

where the second term corresponds to the sum of the cost of all *t*-links that are adjacent to the source and sink vertices *s* and *t*. We omit the contribution of these *t*-links since they can never be selected for a multi-cut due to their high assigned cost.

**Figure 7:**
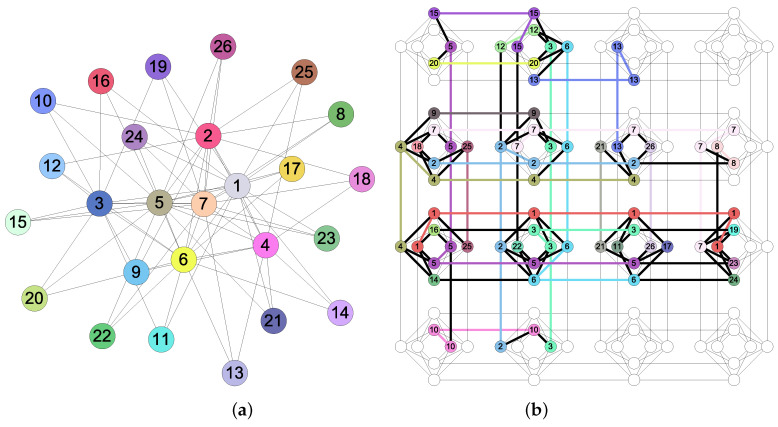
An example of minor embedding: (**a**) logical graph and (**b**) embedding of the logical graph into the hardware of the D-Wave One with 128 physical qubits.

The embedding procedure in step (iv) of the methodology consists of matching the connectivity of a given general graph into the D-Wave graph topology (see Figure 7). Translating a problem expressed as a QUBO function to an identical problem as a subgraph of the graph topology involves a process called minor embedding [56,57]. Commonly, the embedding process requires a number of physical qubits that is greater than the number of logical variables used in a given QUBO expression. Also, finding a minor embedding with the smallest number of physical qubits is an NP-hard problem [57].

The minor embedding problem can be stated as follows. Given the graph topology G of the quantum hardware and a QUBO problem $H_{\mathrm{problem}}^{\mathrm{qubo}}$, represented as a logical graph G=(V,E), where *V* corresponds to the set of Boolean variables in $H_{\mathrm{problem}}^{\mathrm{qubo}}$ and *E* corresponds to the set of edges consisting of a pair of Boolean variables for each quadratic term in $H_{\mathrm{problem}}^{\mathrm{qubo}}$, find a subgraph in G such that

1. Each vertex j∈V is mapped to a connected subtree $T_{j}$ in G.
2. Each edge {i,j}∈E must be mapped to at least one edge in G.

Finding a minor embedding with the smallest number of physical qubits is an NP−hard problem [57]. Figure 7a shows a logical graph with 26 vertices corresponding a QUBO expression, and Figure 7b shows an embedding into the hardware topology of the given logical graph.

We have simulated our algorithm using the software toolbox qbsolv that solves large QUBO problems by partitioning into subproblems targeted for execution on a D-Wave system [58]. Indeed, in addition to qbsolv, there are several QUBO solvers available, among them is [59,60] as well as those methods and software packages mentioned in [61]. We selected qbsolv because we are interested in testing our algorithm on D-Wave’s technology. Source code and full documentation of qbsolv can be found in  [62,63].

Our experiments were run on qbsolv tool using a desktop computer MacBook Air with a 1.3 GHz Intel Core i5 processor and 4 GB of RAM. The motivation of using qbsolv is that if the D-Wave quantum hardware is available, then the problem is partitioned into smaller subproblems that can be minimized by executing a quantum search on the quantum hardware. On the other hand, if no hardware is available, then the qbsolv tool executes the classical Tabu search algorithm to solve each subproblem.

We compare the solutions obtained using qbsolv with a traditional block matching technique. In Figure 4c, we present the truth disparity map which consists of a square region of 7×7 pixels with a depth of 3 pixels. For the BRDS stereo pair, the result using a block matching algorithm can be seen in Figure 4d, and the result obtained using qbsolv can be seen in Figure 4e. In the first case, the square region is partially reconstructed with high disparity values, and in the second case it is almost completely reconstructed with low disparity values. In the latter case, it takes 91.67 s of classic cpu time and 1288 calls using qbsolv. For the gray-scale stereo pair images in Figure 5, we compare the result of a block matching algorithm with sub-pixel accuracy shown in Figure 5c with the result using qbsolv shown in Figure 5d. Figure 5e shows the absolute difference error between the disparity maps in Figure 5c,d, where the mean absolute error is 0.7359 pixels. In this case, it takes 148.31 s of classic cpu time and 1426 calls using qbsolv.

The examples presented in this section were selected with the purpose of showing how our proposal could be implemented. Due to hardware constraints, images used in this section are composed of a limited number of bits. We leave for future work a scalability analysis of our algorithm as well as a comparison study of our algorithm with its classical counterparts.

## 4. Conclusions

We have presented a method for rewriting the stereo matching problem as QUBO expressions based on the graph-cut and minimum multi-cut problems and the Ishikawa reduction method, followed by using QUBO expressions to simulate quantum annealing algorithms on D-Wave’s simulation software. This method has been validated using BRDS and gray scale stereo pairs of images. Future research will be focused on reducing the number of vertices in the graph-cut formulation of the stereo matching problem. This can be achieved by doing an initial disparity search to bound the limits of the disparity range for every pixel of the reference image as well as by incorporating an occlusion term in the cost model given in (11) to penalize occluded regions. An important contribution of this research is the quantum formulation of the minimum multi-cut problem for the case of a single pair of vertices. This formulation consists of the construction of a QUBO expression that is equivalent to the graph-cut problem; hence, no reduction algorithms are needed.

Although the proposed problem can be efficiently solved in a classical way, our methodology provides us with a new way of dealing with problems using quantum-annealing based algorithms and quantum technologies, such as the D-Wave annealer processor, to solve tasks within the realm of machine learning and related areas.

## Figures and Tables

**Table 1:** For case studies (a) and (b) in Figure 4 and Figure 5, respectively, the region of interest (ROI), the disparity range, the dimension of the weighted graph described in Section 2.1.3, and the number of logical variables used to formulate the minimum multi-cut problem given in Equation (19) are shown.

Case	ROI	L	|V|	|E|	${|H}_{\mathrm{problem}}^{\mathrm{qubo}}|$
(a)	15×12	3	902	2745	6392
(b)	15×10	5	1052	3125	7302
